# Video Visits Using the Zoom for Healthcare Platform for People Receiving Maintenance Hemodialysis and Nephrologists: A Feasibility Study in Alberta, Canada

**DOI:** 10.1177/20543581211008698

**Published:** 2021-04-26

**Authors:** Meaghan Lunney, Chandra Thomas, Doreen Rabi, Aminu K. Bello, Marcello Tonelli

**Affiliations:** 1 Department of Community Health Sciences, Cumming School of Medicine, University of Calgary, AB, Canada; 2 Department of Medicine, Cumming School of Medicine, University of Calgary, AB, Canada; 3 Division of Nephrology & Immunology, University of Alberta, Edmonton, Canada

**Keywords:** video visit, virtual care, kidney failure, maintenance hemodialysis, feasibility

## Abstract

**Background:**

Demand for virtual visits (an online synchronous medical appointment between a health care provider and patient) is increasing due to the COVID-19 pandemic. There may be additional benefits of virtual visits as they appear to be convenient and potentially cost-saving to patients. People receiving maintenance hemodialysis require ongoing care from their nephrologist and may benefit from virtual visits; however, the optimal model for a virtual kidney clinic is unknown.

**Objective:**

To codesign and assess the feasibility of a virtual (video) kidney clinic model with clinic staff, nephrologists, and patients receiving maintenance hemodialysis, to be used for routine follow-up visits.

**Design:**

Mixed-methods study.

**Setting:**

Two main kidney clinics in central Calgary, Alberta.

**Participants:**

Adults with kidney failure receiving maintenance hemodialysis, nephrologists, and clinic staff.

**Methods:**

First, we individually interviewed clinic staff and nephrologists to assess the needs of the clinic to deliver virtual visits. Then, we used participant observation with patients and nephrologists to codesign the virtual visit model. Finally, we used structured surveys to evaluate the patients’ and nephrologists’ experiences when using the virtual model.

**Results:**

Eight video visits (8 patients; 6 nephrologists) were scheduled between October 2019 and February 2020 and 7 were successfully completed. Among completed visits, all participants reported high satisfaction with the service, were willing to use it again, and would recommend it to others. Three main themes were identified with respect to factors influencing visit success: IT infrastructure, administration, and process.

**Limitations:**

Patients received training on how to use the videoconference platform by the PhD student, whom also set up the technical components of the visit for the nephrologist. This may have overestimated the feasibility of virtual visits if this level of support is not available in future. Second, interviews were not audio-recorded and thematic analysis relied on field notes.

**Conclusions:**

Video visits for routine follow-up care between people receiving hemodialysis and nephrologists were acceptable to patients and nephrologists. Video visits appear to be feasible if clinics are equipped with appropriate equipment and IT infrastructure, physicians are remunerated appropriately, and patients receive training on how to use software as needed.

## Abrégé

**Contexte:**

La demande pour des consultations virtuelles (rendez-vous médical par vidéoconférence entre un patient et son fournisseur de soins) augmente en raison de la pandémie de COVID-19. Ces consultations pourraient présenter des avantages pour les patients, notamment en raison de leur côté pratique et des économies qui en résultent. Les patients recevant des traitements d’entretien par hémodialyse nécessitent un suivi continu de la part de leur néphrologue et pourraient tirer profit de ce type de consultation. On ignore toutefois quel modèle de clinique de néphrologie virtuelle serait optimal.

**Objectif:**

Concevoir, conjointement avec le personnel des cliniques, les néphrologues et les patients recevant des traitements d’hémodialyse, un modèle de consultation virtuelle (vidéoconférence) pour les visites de suivi de routine, et en évaluer la faisabilité.

**Type d’étude:**

Étude à méthodes mixtes.

**Cadre:**

Les deux principales cliniques de néphrologie du centre de Calgary (Alberta).

**Sujets:**

Des adultes atteints d’insuffisance rénale et recevant des traitements d’hémodialyse, des néphrologues et le personnel des cliniques concernées.

**Méthodologie:**

En premier lieu, le personnel de la clinique et les néphrologues ont été interrogés individuellement afin d’évaluer les besoins de la clinique en matière de consultations virtuelles. Les observations des participants ont ensuite servi à la conception conjointe du modèle de consultation virtuelle avec les patients et les néphrologues. Enfin, des questionnaires structurés ont servi à évaluer les expériences des patients et des néphrologues lors de l’utilisation du modèle.

**Résultats:**

Huit consultations virtuelles (8 patients; 6 néphrologues) étaient prévues entre octobre 2019 et février 2020, dont sept ont été réalisées avec succès. Tous les participants aux séances complétées se sont dits très satisfaits du modèle et ont mentionné qu’ils seraient prêts à l’utiliser à nouveau et qu’ils le recommanderaient à d’autres. Trois principaux thèmes ont été dégagés quant aux facteurs influençant le succès de la consultation ont été dégagés: l’infrastructure, l’administration et le processus informatiques.

**Limites:**

Les patients avaient reçu une formation sur l’utilisation de la plateforme de vidéoconférence de la part d’un étudiant au doctorat, lequel a également mis en place les composantes techniques de la consultation pour le néphrologue. La faisabilité des consultations virtuelles pourrait être surestimée si ce niveau de soutien n’est pas offert à l’avenir. Deuxièmement, les entrevues n’étaient pas enregistrées sur des fichiers audio, l’analyse thématique reposait donc sur des notes d’observation.

**Conclusion:**

Les patients hémodialysés et les néphrologues ont jugé acceptable ce modèle de consultations virtuelles pour les suivis de routine. Les consultations par vidéoconférence sont réalisables si les cliniques sont dotées de l’équipement et de l’infrastructure informatiques appropriés, si les médecins sont rémunérés adéquatement et si les patients reçoivent une formation sur l’utilisation du logiciel, au besoin.

## Introduction

The adoption of virtual care is expanding due to the COVID-19 pandemic. There are many forms and applications of virtual care. Video visits are clinical appointments occurring between a patient and health care provider by video, an alternative to in-person or telephone. While video visits are not appropriate in every circumstance, early reports suggest that overall, they may offer some benefits^1,2^ and should remain integrated within the system in future. While video visit technology today is fairly straightforward, integrating video visits into existing health care systems is challenging. Ensuring the technology is easy to use and functional^3^ is a prerequisite for successful implementation of video visits; however, implementation into a clinical setting presents other challenges. Video visit adoption in practices requires significant change management and ongoing guidance, training, troubleshooting, and support,^4^ that accounts for the unique aspects of each practice setting.^5^

People that receive maintenance hemodialysis frequently meet with a nephrologist for routine follow-up visits. Virtual visits may be appropriate for many of these encounters, as physical assessments are not always required. Our earlier work found that patients, nurses, and nephrologists were interested in trying video visits and reported several theoretical benefits that this mode of care delivery may offer.^6^ However, in addition to their potential benefits, video visits also have potential risks, such as those related to a limited physical assessment or potential disruption to the patient-provider relationhip,^7^ particularly if technology is a barrier to effective communication.^8^ Therefore, broad implementation of video visits will require thorough evaluation of benefits and risks to prevent unintended consequences.

We did this present study to (1) design and test a virtual (video) kidney clinic model for routine visits between patients on hemodialysis and their nephrologist and (2) identify the barriers and facilitators of video visits that may guide future implementation.

## Methods

### Setting

We focused our study at two major kidney clinics in central Calgary, Canada, which is operated by the provincial health authority, Alberta Health Services (AHS). In addition to meeting with nephrologists during dialysis rounds, patients have routine visits 3 to 4 times a year with their primary nephrologist at the kidney clinic. These visits are typically offered in person and occasionally through the provincial Telehealth platform, which still requires patients to travel to a registered health facility to attend the visit. At the time of this study, interest in offering video visits where patients attend from their own homes through simple videoconferencing software was increasing. The Microsoft Skype for Business platform was available through AHS as a video visit platform allowing patients to visit from their own homes, which was scheduled through the AHS Outlook personal information manager. We planned to use Skype for Business software for this study.

### Design

**Figure 1.**
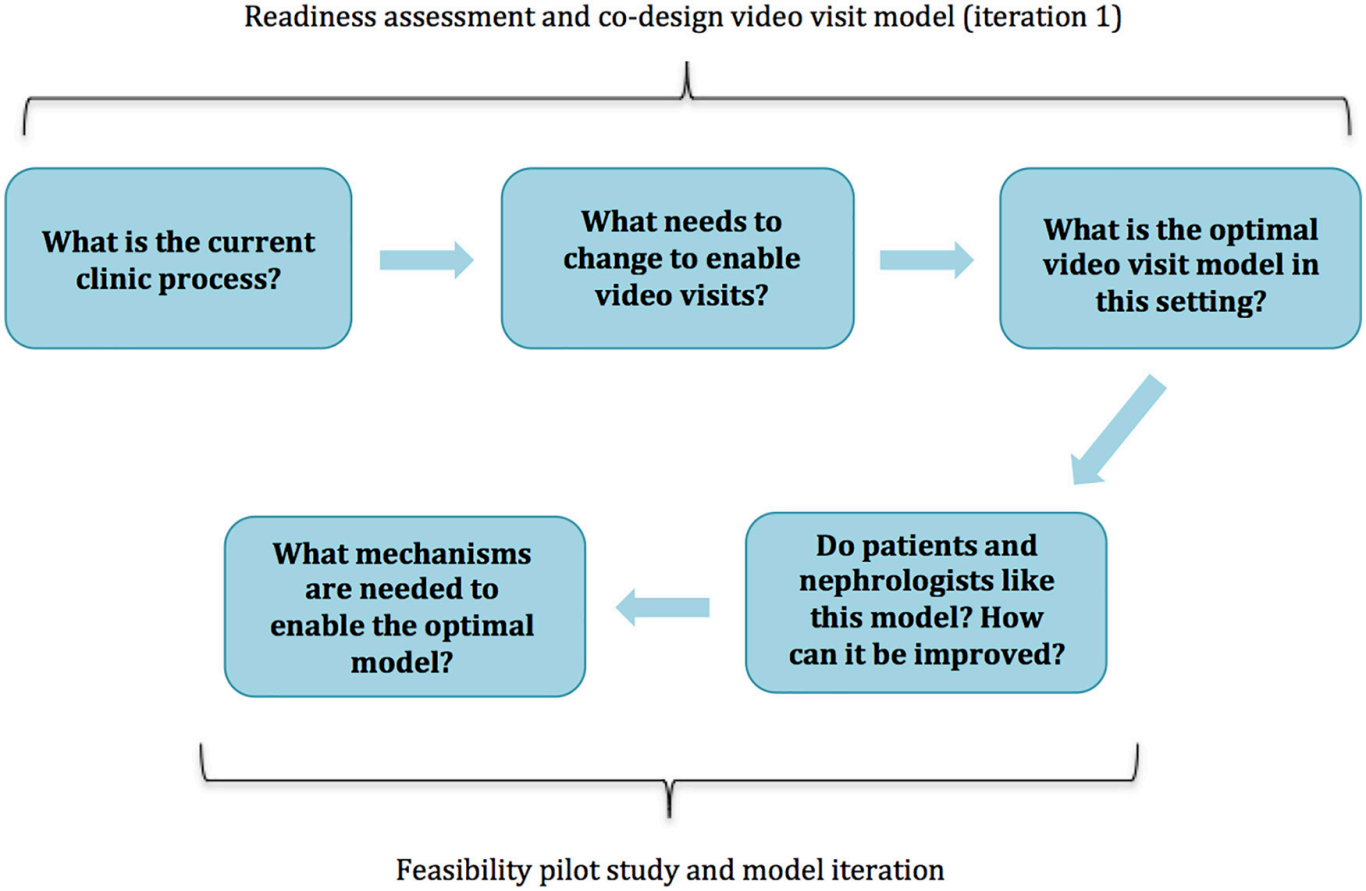
Concept map of research study.

We iteratively designed a video visit model using a mixed methods approach. The main objective was to assess patient and nephrologist readiness and identify process considerations through trial and error and observation (Figure 1). Full methods are described in Supplemental Item 1. First, we assessed clinic readiness through interviewing nephrologists and clinic staff using an equipment and process checklist (Table S1) to identify the logistical requirements needed for video visits in this particular context (*readiness assessment*). Second, we assessed the feasibility of video visits and iteratively redesigned the virtual visit model through observing actual clinical encounters and a structured survey completed by nephrologists and patients (*feasibility pilot study*). The intention of this work was to iteratively trial and redesign the virtual visit model until no new feasibility considerations were identified. Therefore, we did not formally calculate a sample size, but recruited until participant and feedback saturation was met. We did not audio record interviews or code transcripts; however, saturation was determined through comprehensive field notes. The University of Calgary research ethics board approved this study, and the study was in adherence to the Declaration of Helsinki. All participants provided informed consent.

### Recruitment

#### Readiness assessment

Nephrologists holding clinics for maintenance hemodialysis patients at the kidney clinic were approached to participate in the readiness interview (Figure S1). Two staff members from the clinic were purposively sampled to participate due to their role in managing clinical workflow.

#### Feasibility pilot study

We invited nephrologists that participated in the readiness assessment to participate in the pilot study. We supplemented this sample with additional nephrologists to increase the diversity of the study sample. Patients that had previously been interviewed^6^ were invited to participate. In addition, nephrologists that agreed to participate in the feasibility pilot study were asked to identify potential patients, who were contacted by the PhD student (M.L.) if permission was granted.

### Study Process and Procedures

**Figure 2.**
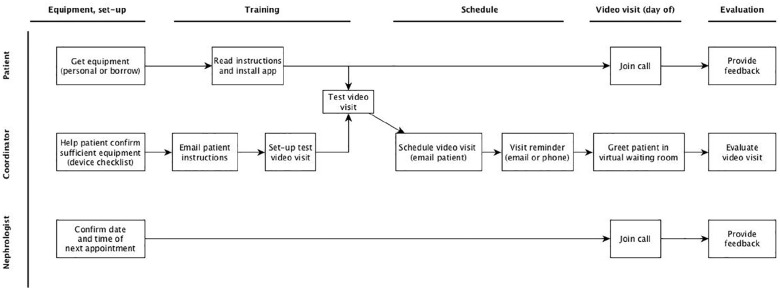
Description of the video visit process tested during the feasibility pilot study.

Full details of the video visit intervention are described in Supplemental Item S1 and presented in Figure 2. In brief, once a nephrologist-patient pair agreed to be in the study, M.L. trained the patient as needed to use the platform independently, met the nephrologist at the clinic, greeted the patient in the virtual waiting room, and then handed the study laptop to the nephrologist to complete the encounter. M.L. met with the nephrologist and patient separately after the visit to collect feedback about the experience using modified existing surveys.^9,10^ Comprehensive field notes were collected during each encounter (training, scheduling, or visit) to capture what factors facilitated or hindered video visits. These field notes were classified into different themes and any new themes identified through subsequent visits were added. Patient-provider pairs were recruited until no new themes were found.

## Results

### Participants

#### Readiness assessment

Seven nephrologists participated in the readiness assessment (see Supplemental Item S1 and Figure S1 for recruitment details). Four (57%) were female, and the median number of years in practice was 14. Six of the 7 nephrologists were remunerated by way of salary and 1 by fee-for-service. Two members of the booking team were purposively recruited to participate in the study. Both agreed to participate; 1 was female and 1 was male.

#### Feasibility pilot study

Three of the 7 nephrologists that participated in the readiness assessment participated in the feasibility pilot study and an additional 3 were invited. Three patients that previously participated in the qualitative study participated and an additional 5 were recruited through nephrologists. In total, 6 nephrologists and 8 patients participated (Table 1). Characteristics of the nephrologists were similar to those participating in the readiness assessment. Among patients, 3 (38%) of the 8 patients were female, the median age was 58 years, 4 (50%) received dialysis at home, 5 lived in a rural area (defined as at least 50 km from the kidney clinic), 1 lived in a nearby city (40 km), and 2 lived in Calgary.

**Table 1.** Demographics of Study Participants in Video Visit Pilot.

Characteristic	N (%) or median (range)
Patients (n = 8)	
Female	1 (12)
Age	58 (38-72)
Location^a^	
Rural	5 (63)
Nearby city	1 (12)
Calgary	2 (25)
Site of dialysis	
In center	4 (50)
Home	4 (50)
Participated in evaluation	
Survey A	5 (63)
Survey B	6 (75)
Interview	8 (100)
Nephrologists (n = 6)	
Female	1 (17)
Years in practice	15 (5-28)
Compensation model	
Fee-for-service	2 (33)
Salary	4 (67)

a Rural = at least 50 km from the kidney clinic; nearby city = located within the Calgary region 40 km from the kidney clinic.

### Findings

#### Readiness assessment

Two main themes were identified with respect to clinic readiness for video visits: technology limitations and scheduling and workflow considerations. Based on our findings from this study and the earlier qualitative interviews,^6^ we developed a video visit intervention with the following features:

- Nephrologists use a laptop at the clinic with a built-in webcam and microphone.
- Have an Ethernet cable as a back-up if the wireless Internet connectivity is poor.
- Provide a tablet for patients who do not have their own device.
- Offer software training to patients as needed, before the day of their actual visit.
- Provide nephrologists with day-of support.

#### Feasibility pilot study

A total of 8 video visits were scheduled, involving 8 patients and 6 nephrologists and 7 were successfully completed. One visit was unsuccessful due to IT issues with the Skype for Business platform (the study laptop was not an AHS-owned device and therefore could not properly launch Skype for Business on the facility’s restricted Internet network). Seven visits involving 7 patients and 6 nephrologists were successfully completed using the Zoom for Healthcare platform.

All of the nephrologists (n = 6) and patients (n = 7) that participated in a successful video visit were interviewed about their experience and asked to complete a survey (Supplemental Tables S2 and S3). Overall, participants reported similar quality between video and in-person visits across most domains. All nephrologists agreed that in-person visits allowed for a better physical assessment but indicated that such assessments are not always required. Some patients noted that if training had not been available, they might have had difficulty using the technology. Patients and nephrologists agreed a video visit was more personal than a telephone call, and some nephrologists additionally noted that some degree of physical assessment was possible through video. All participants would recommend video visits to their peers, under appropriate circumstances. While video visits were convenient and cost-effective for patients, all agreed that in-person visits would still be needed on occasion either for a physical assessment or simply to maintain the patient-provider relationship.

We identified current challenges and barriers of virtual visit delivery and summarized into 3 main themes: IT infrastructure, administration, and process (Table 2). Most patients had a device, however not all, and none of the nephrologists had access to clinic computer equipped for video visits. Remuneration and platform access for video visits was unclear at the time of the study. Some patients were able to use Zoom without training; however, others requested support with installing and using software.

**Table 2.** Main Findings and Recommendations From a Video Visit Pilot for the Outpatient Management of People With Kidney Failure.

Theme	Finding	Recommendation
IT infrastructure		
Hardware	Health care providers often do not have computers with built-in webcams or microphones at their clinics.	Allow health care providers to use their own devices or acquire a clinic laptop and share as appropriate.
	Not all patients have devices that work with virtual meeting platforms.	Offer tablets to patients to borrow as needed.
Software	Skype for Business currently experiences issues with Apple products and does not work with iPads. Microsoft is also retiring the platform (or switching to Teams) in the future.	Select a brand and device-agnostic video visit platform.
Internet	Health care providers are unable to use devices not owned by the local health authority (Alberta Health Services) on the restricted network.	Allow devices not owned by Alberta Health Services to use the restricted network for video visits.
	Some patients experienced breaks in video, delays, and so on due to poor Internet connectivity.	This did not appear to be a significant problem. Include in evaluation framework to monitor.
Administration		
Remuneration	The fee codes in Alberta do not currently allow for video visits (at the time of study).	Reassess the remuneration policies and the impact on virtual care delivery. Monitor the impact on system costs.
Governance	There are a number of activities relevant for operationalizing, monitoring, and evaluating virtual visits that need to be owned and managed.	Stakeholders meet, discuss, and individualize.
Process		
Clinical workflow	New roles and responsibilities have been created for clinic staff and need to be clarified.	Identify current capacity in clinic and delegate or hire new staff as appropriate.
	The clinic workflow activities (eg, greeting patient in waiting room, medication reconciliation, notifying patient if provider is running behind, responding to no-shows or late/early arrival, involving caregivers and multiple health care providers, etc) will still need to happen virtually and a revised clinic process for video visits is needed.	Process mapping and change management involve all team members when designing and testing strategy. Implement slowly and in a phased approach and monitor progress and unintended consequences (ie, Plan-Do-Study-Act).
	Virtual and in-person visits should be used together as part of a seamless and integrated system, where the modality of the visit should be intentionally chosen appropriately, considering the unique circumstances and preferences of each encounter.	There should be a formal process for clinics to follow when deciding whether an in-person or video visit is appropriate, which should involve shared decision-making between providers and patients.
Training	Patients will need some training and support with how to use platform properly.	Ensure patients are familiar with how to install and use software. The level and type of support required will vary across patients. Involving family members may help address technology barriers.
	Clinic staff and health care providers should have support with platform.	Employ a train-the-trainer approach in clinics and develop a process of support for clinics and providers as needed.

## Discussion

The goal of this study was to design and test a video visit model and identify the factors needed for success. We tested and iteratively refined the model during 8 encounters involving 6 nephrologists and 8 patients and completed 7 successful visits using the Zoom for Healthcare platform. Among these 7 visits, most patients and nephrologists did not perceive video visits to harm communication or the interaction. One patient felt that the technology made it more difficult to connect with the doctor than an in-person visit and believed that the technology may negatively affect their communication. Not surprisingly, all nephrologists agreed that in-person visits allow for a better physical examination. All participants agreed that video visits should not completely replace in-person visits, but that supplementing in-person care with video visits when appropriate would improve convenience and access for patients.

Feasibility studies in other settings have reported similar results. Patients appear to enjoy the convenience of being able to attend visits from the comfort of their own home, saving them time and money.^1,11-15^ This may be particularly useful for specialist care, as in Canada, specialist clinics are typically located in cities and people living in rural areas are less likely to consult a specialist physician.^16^ In addition, virtual visits allow patients to safely continue accessing care during the COVID-19 pandemic, which is important as halting the follow-up of maintenance conditions can have devastating downstream effects for patients and the health care system.^17^

Our study provided patients with training on how to install Zoom software on their devices and use the platform. We also did a test run with each patient before their actual visit to ensure that they were able to use Zoom independently. This support likely increased the success rate of video visits, and also appeared to empower patients.^18^ As virtual care becomes more integrated in our health care system, ensuring patients are equipped with the tools and skills to participate is important, particularly to avoid inequity^19^ as not everyone has the same access to or experience with technology.

To move forward with virtual care, clinics need to determine the changes needed to implement virtual care, which will be specific to each setting. Checklists^20^ that include IT; scheduling processes; provider, staff, and patient training; and electronic medical record (EMR) integration; among others may help prepare clinics for implementation. Provincial and national administration and policies are unclear. A national virtual care taskforce in Canada has proposed 19 recommendations to move forward with virtual care,^21^ which include national standards, safety and quality framework, remuneration, education and curriculum for providers, and licensing, among others. To ensure consistent, safe, and high-quality virtual care, future efforts that focus on the administration, governance, and health information systems of virtual care are needed.

The findings from our study provide practical considerations for the future implementation of video visits in Alberta. In brief, clinics should be equipped with sufficient IT infrastructure that enables high-quality video visits. Patients should receive training on an as-needed basis and have an option to borrow a tablet if they do not have access to 1 of their own. Clinic processes will need to be adapted to allow for the integration of video visits in practice, such as scheduling, greeting patient upon arrival, and collecting information to prepare the chart for the nephrologist for the visit. Where clinical data are not available during the visit (such as in an EMR system), designing a process for dialysis nurses and clinic staff to communicate and share information before the visit will be helpful, particularly for home dialysis patients that often bring hard copy material to the appointment.

### Limitations

These findings should be interpreted within the limitations of the study. First, our work focused on the episodic success of video visits and did not follow up patients to explore efficacy outcomes. It is possible that even though virtual visits often save patients’ travel-related time and costs, they may not be clinically appropriate in all circumstances and could lead to negative effects. Second, the PhD student (M.L.) was involved in the intervention delivery. She trained patients on how to use the platform and participated in visit activities the day of the appointment (met patients in the waiting room, connected the nephrologist to the call). While this offered important insight through participant observation, involving clinic staff in this process may have yielded helpful information to better understand how the clinic process needs to adapt to accommodate for these new duties. Third, our work aimed to identify clinic readiness for video visit implementation and factors influencing the success of virtual visits. Both these parameters are context specific, and our findings may not apply to other settings. Finally, the pilot study occurred before the COVID-19 pandemic and it is possible that several of the recommendations based on our findings have been already addressed.

## Conclusions

Video visits for follow-up routine encounters between nephrologists and people on hemodialysis are feasible and acceptable, given sufficient technology and patient training are available. Virtual visits should be integrated into the health care system and used to complement in-person care when appropriate. Scheduling processes in the clinic will need to be updated to accommodate video visits. Ownership of duties related to video visit setup, patient training, and supporting nephrologists with any technical issues needs to be clarified, as does governance and medical-legal policies. Leveraging the EMR and increasing communication between dialysis site staff and nephrologists may help improve the effectiveness of video visits.

## Supplemental Material

sj-pdf-1-cjk-10.1177_20543581211008698 – Supplemental material for Video Visits Using the Zoom for Healthcare Platform for People Receiving Maintenance Hemodialysis and Nephrologists: A Feasibility Study in Alberta, Canada Click here for additional data file. Supplemental material, sj-pdf-1-cjk-10.1177_20543581211008698 for Video Visits Using the Zoom for Healthcare Platform for People Receiving Maintenance Hemodialysis and Nephrologists: A Feasibility Study in Alberta, Canada by Meaghan Lunney, Chandra Thomas, Doreen Rabi, Aminu K. Bello and Marcello Tonelli in Canadian Journal of Kidney Health and Disease
