# Supplementary material for: Video Visits Using the Zoom for Healthcare Platform for People Receiving Maintenance Hemodialysis and Nephrologists: A Feasibility Study in Alberta, Canada
Source: Can J Kidney Health Dis. 2021 Apr 26;8:20543581211008698. doi: 10.1177/20543581211008698 (PMC8082995; doi:10.1177/20543581211008698)
Supplement: sj-pdf-1-cjk-10.1177_20543581211008698 – Supplemental material for Video Visits Using the Zoom for Healthcare Platform for People Receiving Maintenance Hemodialysis and Nephrologists: A Feasibility Study in Alberta, Canada [file sj-pdf-1-cjk-10.1177_20543581211008698.pdf]

Supplemental material

Item S1. Full methods and recruitment details.

First, we interviewed nephrologists and kidney clinic staff to assess clinic readiness and identify the logistical requirements needed for video visits in this particular context (*readiness assessment*). Nephrologists were interviewed individually and clinic staff were together, both using a guide (Supplemental Table S1). We had previously interviewed patients, nurses, and nephrologists about using video visits in their care[1] and also included this input during initial design. This phase informed the first design draft of the video visit model.

Second, we explored the feasibility of this model. The goal was not to implement the model, but understand what mechanisms were needed to enable delivery and further, iterate the design as needed based on practical experience. We assessed the feasibility of video visits through exploring platform usability and process requirements through actual clinical encounters (*feasibility pilot study*). We measured patient and nephrologist experience and iteratively redesigned the process after each encounter.

Readiness assessment

Nephrologists holding clinics for maintenance hemodialysis patients at a major kidney clinic in Calgary, Alberta were approached to participate in a short interview. Twenty-two potentially eligible nephrologists were identified. Of these, 16 (80%) received an email to inform them about the feasibility study and offer an opportunity to participate. Two nephrologists had missing contact information, one was a co-investigator in the study, and one was approaching retirement and therefore did not receive an invitation to participate. Of the 16 nephrologists that received an invitation, 7 (44%) agreed to be interviewed. Nephrologists were asked about their access to information technology (IT) equipment and their current workflow of conducting clinic visits. Two staff members from the clinic were

[1] Lunney M, Finlay J, Rabi DM, et al. eVisits in Rural Hemodialysis Care: A Qualitative Study of Stakeholder Perspectives on Design and Potential Impact to Care. American Journal of Kidney Diseases. 2020 Sep;76(3):441-444

also invited to participate due to their role in managing clinical workflow. They were asked about the current scheduling process and to offer suggestions on how video visits could be incorporated into the process. The PhD student (ML) interviewed all participants using a guide (Table S1). Interviews were not recorded or transcribed but ML took field notes and produced narrative summaries of findings.

Feasibility pilot study

We invited the 7 nephrologists that participated in the readiness interviews to participate in the pilot study (Figure S1). Three agreed to participate. Of the 4 that declined, 2 were not seeing any outpatients in Calgary during the study period and 2 could not identify any patients that, in their opinion, were appropriate for testing video visits (i.e., not clinically stable). Four additional nephrologists were purposively invited either because patients under their care were interested in participating (n=2) or to make the sample more gender-balanced (n=2). Three agreed to participate. In total, 6 nephrologists agreed to participate in the feasibility pilot. Patients that had previously been interviewed[1] were invited to participate. Of the 11 patients that participated in the interviews, 4 were not eligible for the pilot study (one had passed away, 2 were hospitalized, and one had moved to another site). Of the 7 eligible patients, 4 (57%) were not interested. To increase study participation, nephrologists that had agreed to participate were also asked to identify additional potential participants, whom were contacted by the PhD student if permission was granted. Four nephrologists identified a total of 5 patients. All 5 of these patients agreed to participate. In total, 6 nephrologists and 8 patients participated in the pilot (2 nephrologists participated twice with 2 different patients).

Once a nephrologist-patient pair agreed to participate, the PhD student (ML) contacted patients to assess training needs. Patients were emailed information about the study as well as instructions on how to install the software and use the platform. ML either met with patients in-person (n=5) to observe them attempting to set-up the platform or had a follow-up call over phone or video (n=3) to inquire about usability. Once patients received training and completed a test-run successfully, ML emailed them a reminder a day before the appointment with the link to the visit. On the day of, ML met the nephrologist at the

clinic, greeted the patient in the virtual waiting room, ensured audio and visuals were working, and passed the laptop to the nephrologist for the encounter. ML was not in the clinic room during the visit, but was available in the clinic if issues arose. After each encounter, ML interviewed nephrologists and patients individually to learn about their experience and each participant completed a satisfaction survey (Supplemental Tables S2-S4). Interviews were not recorded or transcribed but ML took field notes and produced narrative summaries of findings.

Initially, we chose the Skype for Business software for the video visit platform as this was offered by Alberta Health Services (AHS) for video visits at the time of the study. The platform is integrated with Microsoft Outlook, from which video visits can be booked directly. However, in July 2019, Microsoft announced it would be retiring the platform and all users would be migrating to Microsoft Teams. At this time we had already experienced a number of technological issues with the Skype for Business plug-in, particularly when used on Apple products, so decided to find an alternative platform. Alberta was nearing a provincial roll-out of an electronic clinical information system (Connect Care) and the Zoom for Healthcare (Zoom Video Communications, Inc.) platform integrates with the Connect Care platform. Further, the Zoom for Healthcare product is additionally compliant with HIPAA and although this law does not apply to Alberta, it ensures a standard of data privacy and health information protection. Therefore, we decided to switch to the Zoom for Healthcare platform for the remainder of the study.

Table S1. Interview guide for readiness assessment.

<table>
<tr><th>Nephrologists</th></tr>
<tr><td>Technology

Do you have access to an Alberta Health Services’ computer in your clinic room with a webcam and microphone?

(if yes) Does this computer:
• have Microsoft Outlook 2013 (or higher)?
• run on Windows 7 or higher?
• run on Windows XP?

Administration

Do you have access to a secretary whose computer has access to the AHS network?

Who typically schedules the follow-up visits for patients receiving maintenance hemodialysis? What is that process and how would it need to be adapted for video visits?

Interest

If one of your patients were interested in using video visits, would you consider trying the platform when appropriate (recognizing that some situations do require an in-person visit)?

If one of your patients preferred to book a video visit outside of your regular clinic hours, would you consider this? Note: video visits do not require a nurse to be present.

Are you paid through a fee-for-service contract or salary?

Do you have any final comments?</td></tr>
<tr><th>Clinic staff</th></tr>
<tr><td>Clinic staff were provided instructions on how to schedule a Skype for Business visit using Outlook and asked the following questions:

Did you experience any problems with scheduling the visit in Outlook? If so, what were they? What are your suggestions to make this easier?

Were the instructions clear on how to create a link for a visit? If no, please explain which steps were unclear. How could they be better?

Clinic staff were provided an example of instructions for patients and asked for input on the content and process:

What information do you currently send to patients for in-person visits? How might this</td></tr>
</table>

change for a video visit?

Is anything missing on these instructions that needs to be considered?

Clinic staff were asked a series of questions about general workflow:

What is the current process if patients want to reschedule their appointments? Could this be done easily with video visits?

If patients had an in-person visit and wanted to change to a video visit (or visa versa), what process do you suggest?

What support is currently available through Telehealth? What support do you think would be needed for Skype for Business?

Do you have any final comments?

Table S2. Patient and nephrologist perspectives of video visit quality, compared to in-person visits.

	Better in-person	Better in video visit	No difference
**Patient (n=5)[1]**			
I felt reassured	0	0	5
I felt that I was taken care of	0	0	5
We had a good talk	0	0	5
The doctor understood what was on my mind	1	0	4
I was happy with the amount of time spent on small talk	0	0	5
It was a bit difficult to ask questions	0	0	5
Important decisions were made over my head	1	0	4
It was a bit difficult to connect with the doctor	1	0	4
**Nephrologist (n=5)[2]**			
I felt that the patient was reassured	0	0	5
I felt that the patient was taken care of	0	0	5
We had a good talk	0	1	4
I understood what was on the patient's mind	0	0	5
I was happy with the amount of time spent on small talk	0	2	3
It was a bit difficult to ask questions	0	0	5
Important decisions were made over the patient's head	1	0	4
It was a bit difficult to connect with the patient	0	0	5
I was able to physically assess the patient	5	0	0

Survey adapted from the framework used by Liu et al.[10]

[1] Three patients didn't complete the survey: one patient wasn't asked to complete the survey as the video visit wasn't completed due to Internet failure at clinic (using the Skype for Business platform); one patient filled out an earlier version of the survey that didn't compare video visits to in-person visits; one patient did not complete the online form.
[2] One nephrologist completed an earlier version of the survey, which didn't compare video visits with in-person visits.

Table S3. Patient and nephrologist reported acceptability of video visits and user satisfaction.

	Agree completely	Agree	Neither agree nor disagree	Disagree	Disagree completely
**Acceptability**					
**Patient (n=6)**[1]					
Using the technology was easy for me	5	1	0	0	0
I was able to see my doctor	6	0	0	0	0
I was able to hear my doctor	6	0	0	0	0
The quality of the video visit (technology only) was good	5	1	0	0	0
**Nephrologist (n=6)**					
Using the technology was easy for me	5	1	0	0	0
I was able to see the patient	5	1	0	0	0
I was able to hear the patient	5	1	0	0	0
The quality of the video visit (technology only) was good	5	1	0	0	0
**Satisfaction**					
	Very satisfied	Fairly satisfied	Neither satisfied nor dissatisfied	Fairly dissatisfied	Very dissatisfied
**Patient (n=6)**[1]					
Overall, how satisfied or dissatisfied were you with using the video visit service for your health assessment?	5	1	0	0	0
**Nephrologist (n=6)**					
Overall, how satisfied or dissatisfied were you with using the video visit platform for assessing the health of the patient?	6	0	0	0	0
	Extremely likely	Likely	Neither likely nor unlikely	Unlikely	Extremely unlikely
**Patient (n=6)**[1]					
How likely are you to recommend the video visit service to friends and family if they need similar care or advice?	6	0	0	0	0
**Nephrologist (n=6)**					
How likely are you to recommend the video visit service to other physicians if they need to provide similar care?	6	0	0	0	0

Acceptability survey adapted from the framework used by Liu et al.[10]
Satisfaction survey adapted from the framework used by Cowie et al.[9]

[1] Two patients didn't complete the survey: one patient wasn't asked to complete the survey as the video visit wasn't completed due to Internet failure at clinic (using the Skype for Business platform). One patient did not complete the online form.
